# Including Methane Emissions from Agricultural Ponds in National Greenhouse Gas Inventories

**DOI:** 10.1021/acs.est.3c08898

**Published:** 2024-05-02

**Authors:** Martino E. Malerba, Tertius de Kluyver, Nicholas Wright, Odebiri Omosalewa, Peter I. Macreadie

**Affiliations:** † Deakin Marine Research and Innovation Centre, School of Life and Environmental Sciences, Deakin University, Melbourne, Victoria 3125, Australia; ‡ Energy, The Environment and Water, Emissions Reduction Division, Australian Department of Climate Change, Canberra, Australian Capital Territory 2601, Australia; § Department of Primary Industries and Regional Development, Sustainability and Biosecurity, 1 Nash St, Perth, Western Australia 6000, Australia

**Keywords:** farm dams, agricultural ponds, agricultural reservoirs, impoundments, dugouts, eutrophication, inland wetlands, methane ebullition, national inventory reports

## Abstract

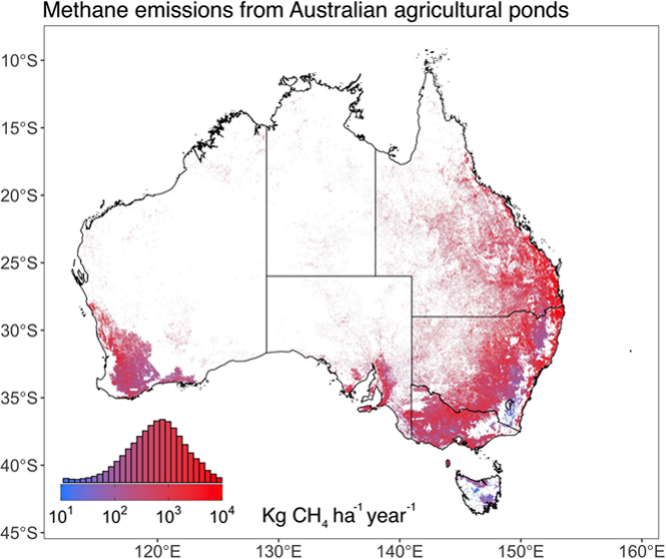

Agricultural ponds are a significant source of greenhouse gases, contributing to the ongoing challenge of anthropogenic climate change. Nations are encouraged to account for these emissions in their national greenhouse gas inventory reports. We present a remote sensing approach using open-access satellite imagery to estimate total methane emissions from agricultural ponds that account for (1) monthly fluctuations in the surface area of individual ponds, (2) rates of historical accumulation of agricultural ponds, and (3) the temperature dependence of methane emissions. As a case study, we used this method to inform the 2024 National Greenhouse Gas Inventory reports submitted by the Australian government, in compliance with the Paris Agreement. Total annual methane emissions increased by 58% from 1990 (26 kilotons CH_4_ year^–1^) to 2022 (41 kilotons CH_4_ year^–1^). This increase is linked to the water surface of agricultural ponds growing by 51% between 1990 (115 kilo hectares; 1,150 km^2^) and 2022 (173 kilo hectares; 1,730 km^2^). In Australia, 16,000 new agricultural ponds are built annually, expanding methane-emitting water surfaces by 1,230 ha yearly (12.3 km^2^ year^–1^). On average, the methane flux of agricultural ponds in Australia is 0.238 t CH_4_ ha^–1^ year^–1^. These results offer policymakers insights into developing targeted mitigation strategies to curb these specific forms of anthropogenic emissions. For instance, financial incentives, such as carbon or biodiversity credits, can mobilize widespread investments toward reducing greenhouse gas emissions and enhancing the ecological and environmental values of agricultural ponds. Our data and modeling tools are available on a free cloud-based platform for other countries to adopt this approach.

## Abstract 2

We developed satellite tools to estimate methane emissions from agricultural ponds in national inventories, aiding policy efforts to address climate change.

## 1 Introduction

**Figure 1:**
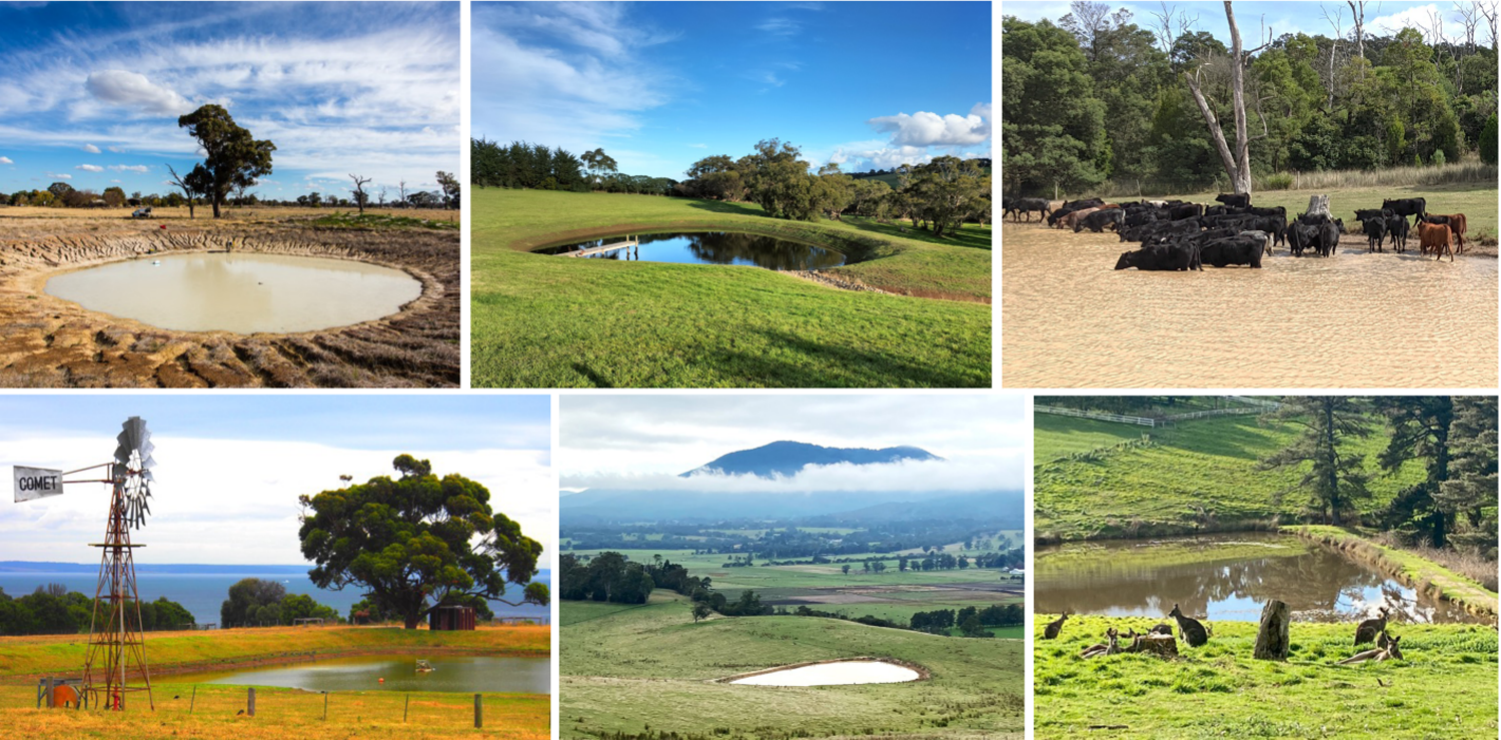
Agricultural ponds (also known as farm dams, agricultural reservoirs, impoundments, or dugouts) are small artificial freshwater systems to store water for livestock and crops. Photos 1–3 (top): reproduced with permission by Dr I. N. Yilmaz. Photos 4–6 (bottom): taken by the author.

Methane concentrations in the atmosphere have increased rapidly and now contribute to approximately a third of anthropogenic climate change.^1^ Methane in today’s atmosphere is over 1,800 ppb, triple the concentration in the early 1900s.^2,3^ Nearly half of all global methane emissions are from aquatic habitats, including artificial waterbodies, such as reservoirs, ponds, and canals.^4^ In particular, agricultural ponds (also known as farm dams, agricultural reservoirs, impoundments, or dugouts; Figure 1) are one of the most abundant artificial waterbodies, covering approximately 7500 kilo hectares (kha; 75,000 km^2^) worldwide.^5^ Despite being small (typically 0.1–1 ha; 1,000–10,000 m^2^), they have some of the highest methane emissions among man-made aquatic systems.^6,7^ Although likely to emit a significant amount of methane, emissions from small waterbodies such as agricultural ponds are frequently neglected in national carbon accounting.^8^

The importance of monitoring methane emissions from agricultural ponds in National Greenhouse Gas Inventory Reports (hereafter “national GHG inventories”) is increasingly recognized.^8^ Managed by the United Nations Framework Convention on Climate Change (UNFCCC), national GHG inventories are critical for tracking anthropogenic emissions and a country’s economic progress toward decarbonizing.^9^ Under the UNFCCC, all participating Annex-I countries submit annual inventories documenting their anthropogenic greenhouse gas emissions and removals, whereas non-Annex-I countries submit biennial update reports. The Intergovernmental Panel on Climate Change (IPCC) provides standard guidelines to develop these reports, enabling comparisons among countries.^10^

The IPCC recently updated its guidelines to include methane emissions from agricultural ponds in their national GHG inventories.^11^ Depending on resources and available data, IPCC guidelines offer three approaches to estimating methane emissions for agricultural ponds,^11^ with their increasing complexity designed to reduce the uncertainty in estimating methane emissions. The simplest approach (tier 1) involves multiplying the cumulative surface area of agricultural ponds by a constant methane emission factor. A more detailed approach (tier 2) requires on-site measurements to calculate country- or region-specific estimates for pond emissions based on local climate conditions. The most complex approach (tier 3) must address local aspects influencing methane emissions, including soil type and land use activities around agricultural ponds.

The methods devised by IPCC for agricultural ponds have been derived from existing techniques used for larger waterbodies such as reservoirs. However, their application to smaller systems, such as ponds, presents three main limitations. First, many agricultural ponds are ephemeral and their surface water fluctuates substantially between dry and wet seasons, complicating the calculations on the methane-releasing surface area.^12,13^ Second, agricultural ponds are shallower and warm up faster than larger waterbodies, needing additional steps to capture the temperature sensitivity of methane production.^14,15^ Third, national GHG inventories require a time series starting from 1990, predating the establishment of many ponds. Yet, data on the accumulation rate of agricultural ponds are scarce and often subject to errors.^16^ Overlooking the effects of the water surface, temperature, or pond accumulation will likely have important implications for yearly estimates of methane emissions from agricultural ponds. Refining current methodologies by addressing these limitations will help to reduce uncertainties in global anthropogenic methane emissions.

**Figure 2:**
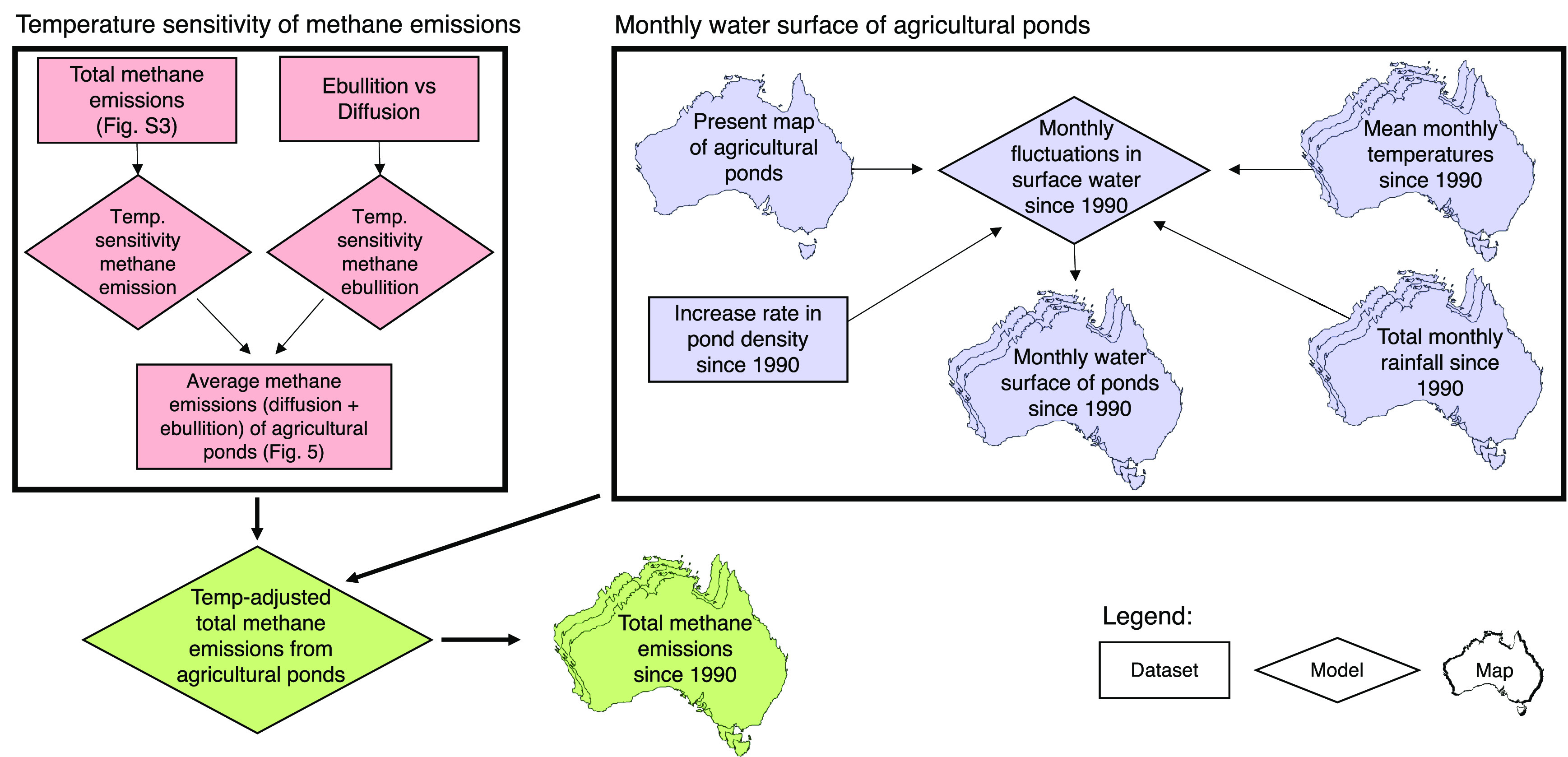
Visual summary of our modeling approach to estimate methane emissions of agricultural ponds used to inform the 2024 national greenhouse gas inventory report of Australia. Rectangles identify datasets, shapes of Australia indicate maps or time series of maps, and diamonds are statistical models. The colors identify the different themes: methane temperature dependency (red), physical footprint and climatic variables (purple), and overall results (green).

This study introduces a novel method to estimate methane emissions from agricultural ponds that accounts for historical changes in pond densities and seasonal fluctuations in temperature and surface water. Our method informed the 2024 edition of Australia’s National Greenhouse Gas Inventory,^17^ and it uses high-resolution satellite datasets to estimate the density, distribution, historical trends, and local climate conditions of Australian agricultural ponds (Figure 2). The method corrects methane emissions for monthly local temperatures using three meta-analyses (red shapes in Figure 2) and quantifies monthly changes in the water surface at each pond using remote sensing (purple shapes in Figure 2). Here, we show the importance of these improvements by comparing estimates from previous national GHG inventories against our results.

## 2 Methods

### 2.1 Agricultural Ponds in Australia

#### 2.1.1 Present Density of Agricultural Ponds

The most recent census of agricultural ponds in Australia estimated 1.76 million ponds covering a maximum area of 467.8 kilo hectares (kha; 4,678 km^2^) and storing up to 10,990 gigaliters (GL).^16^ These statistics are for 2021 and are based on a deep-learning convolutional neural network trained to detect agricultural ponds using high-resolution (normally 0.45 m) red, green, and blue (RGB) satellite images. The training data set was developed from State and Federal maps reporting agricultural ponds across Australia, with agricultural dams being a specific category.^16^ The average surface area of Australian ponds is around 0.1 ha (10^3^ m^2^), ranging from 0.01 to >10 ha (100 to >10^5^ m^2^). These statistics are calculated after excluding ponds larger than 10 ha (10^5^ m^2^) and those that appeared of natural origins (e.g., complex shapes, jiggered borders) by retaining only those with simple and regular shapes, calculated as circularity (4 × area × [π × perimeter^2^]^−1^) above 0.5. The final data set had no duplicate or overlapping shapes and is available online in a free interactive portal at www.AusDams.org. See Malerba, Wright, and Macreadie^16^ for technical details on the training and calibration of the convolutional neural network.

#### 2.1.2 Rates of Increase in Agricultural Pond Densities

We sourced the rates of increase in agricultural ponds from Malerba, Wright, and Macreadie.^16^ The study reports accumulation rates in the density of agricultural ponds in Australia from 1988 to 2015 using spatial layers from the Water Observations from Space (WOfS)^18^ and the Digital Earth Australia Waterbodies (DEAW).^19^ WOfS uses Landsat 5 and Landsat 7 satellite images to detect surface water at a 30 m grid size across Australia at an approximate biweekly frequency. The DEAW elaborates data from WOfS to provide 28 years of biweekly time series of relative wet surface area for 300,000 waterbodies across Australia.^19,20^ The authors identified agricultural ponds by looking for overlapping waterbodies between DEAW and the map of agricultural ponds from^16^ (see Section 2.1.1). The year of establishment of an agricultural pond was the first year when water was consistently reported in at least 25% of the pond’s surface area. Because of the coarse grid size, DEAW time series could only detect the year of establishment of agricultural ponds larger than at least three Landsat pixels (i.e., 0.27 ha or 2,700 m^2^). Without data for smaller ponds, we assumed that smaller agricultural ponds increased at equal relative rates to larger ones (i.e., 1–4% annual increase).^16^

We used establishment dates to calculate relative and absolute rates of pond accumulation in each State and Territory (Figure S1). The only exception was for the Northern Territory, where WOfS reported too few agricultural ponds to calculate a representative rate of increase for the region. Hence, for this region, we used the national average rate of the increase of agricultural ponds. Also, because the available data on historical trends in Malerba, Wright, and Macreadie^16^ were only until 2015, we projected pond densities between 2016 and 2021 using the average annual rates for each State and Territory between 2010 and 2015 (Figure S1).

#### 2.1.3 Pond Surface Area and Maximum Water Surface

We used the models developed by Malerba, Wright, and Macreadie^13^ to measure the surface area of a pond (ha) and its theoretical maximum water surface area (ha), which includes the bare clay area above the waterline. The approach relied on deep-learning convolutional neural networks developed with the Python open-source library “fastai” to analyze 148,344 randomly selected agricultural ponds in Australia (nearly 10% of the total) using RGB satellite images (usually 0.5 m resolution) with acquisition dates between Jan 2011 and Dec 2020 from https://server.arcgisonline.com. The data set included agricultural ponds in the States and Territories of New South Wales (*N* = 36,027), Victoria (*N* = 27,692), Queensland (*N* = 17,884), Western Australia (*N* = 15,789), South Australia (*N* = 5,272), Tasmania (*N* = 4,178), and the Australian Capital Territory (*N* = 61). The Northern Territory had too few images of agricultural ponds, so we used the average pond size in Australia to estimate the total water surface of agricultural ponds in this region. The convolutional neural networks started from pretrained ResNet-18 UNets, followed by further training using manually traced agricultural ponds from 569 randomly selected images using an 80:20 split for both the training and validation data sets. Each training image consisted of a binary mask, with 1 representing the area of interest (either surface water or the maximum fill area of the dam) and 0 representing the background area. The training started in a “frozen” state to speed up computational times for transfer learning (batch size of 8 for 30 epochs at a learning rate of 0.001), followed by 18 more epochs of training in an unfrozen state at a 10-fold lower learning rate (0.0001). All outputs from the convolutional neural networks were satellite images converted into binary masks and measured for the water surface area and total pond area (both in ha). The final cross-entropy losses were 0.064 for tracing surface water and 0.175 for tracing the total pond area. For details, see Malerba, Wright, and Macreadie.^13^

#### 2.1.4 Monthly Time Series of Pond Surface Area

The surface area of agricultural ponds in Australia fluctuates significantly between dry and wet seasons, influencing aquatic methane emissions. To estimate the methane-emitting water surface of a pond without acquiring and processing time series of commercial high-resolution satellite images, we developed an Extreme Gradient Boosting (XGBoost) regression.^21^ We used the XGBoost to predict the water surface area of a pond (ha) using the maximum theoretical pond area (ha; Section 2.1.3) and its local conditions of mean temperature (°C) and cumulative rainfall (mm) in the previous 13 months. We used XGBoost to predict the monthly time series of each agricultural pond in Australia from 1990 to 2022 and calculate nationwide statistics on the total water surface (units of kha).

For the training and validation data sets of the XGBoost, we randomly selected 122,360 high-resolution (0.5 m) RGB satellite images from across Australia acquired between Jan 2011 and Dec 2020. For each image, we first used a classification convolutional neural network to ensure the presence of a pond. If confirmed, we determined the water surface area (ha) and the theoretical maximum water surface area (ha) using the deep-learning convolutional neural network described above (see Section 2.1.3). For this analysis, we excluded ponds larger than 2 ha, as their low frequencies (1.2% of the total) reduced the ability of the model to capture their dynamics. For each image in the training and validation data sets, we compiled monthly data for mean temperature (°C) and cumulative rainfall (mm) for the 13 months before the image acquisition date using ANUClimate v2 monthly gridded data set at a 0.01° grid size.^22,23^ This data set offers historical records since 1965 of several climate variables from climate stations of the Australian Bureau of Meteorology analyzed with the ANUSPLIN package.^24^ Data from ANUClimate v2 allowed us to characterize each agricultural pond with individual time series of local rainfall and temperature. Using 13 months of climate data in the model ensured reliable replication without including events too old to affect current water levels.

For tuning the hyperparameters, we used the Python library “Bayesian optimisation” (“bays_opt”)^25^ to determine optimal learning rates (from 0.1 to 0.5), the number of estimators (from 10 to 700), and the maximum tree depths (from 1 to 10). We trained the models using 15 random combinations of hyperparameters and a further 30 optimized combinations. We selected the top 10 models based on the mean absolute error (MAE) of the validation data set. The 10 models were assembled using soft voting.

The full training and validation data set included 120,939 randomly sampled satellite images of agricultural ponds across Australia. Each image had information about the water surface area, maximum theoretical water surface (using the methods in Section 2.1.3), and 13 months of monthly local conditions for temperature and rainfall before the image acquisition date (using ANUClimate v2). We randomly divided the compiled data set into training (90%) and validation (10%) subsets. Using the XGBoost Python library,^21^ we trained an XGBoost regression model to predict the water surface area based on the climate and pond variables described above. Finally, we further validated model predictions of the XGBoost using a second independent data set of 381 high-resolution satellite images of agricultural ponds from 2009 to 2023 where the water surface areas were manually traced.

### 2.2 Temperature-Dependent Methane Emissions from Agricultural Ponds

We used the Boltzmann–Arrhenius relationship calibrated in Malerba et al.^8^ to estimate the methane flux (including diffusive and ebullitive fluxes) of an agricultural pond in Australia after accounting for temperature, as
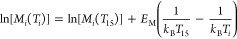
1where ln[*M*_*i*_(*T*_*i*_)] is the log_e_-transformed rate of yearly methane emissions (units of t CH_4_ ha^–1^ year^–1^) at the agricultural pond *i* with local air temperature *T*_*i*_ (in Kelvin), ln[*M*_*i*_(*T*_15_)] is the average total yearly methane emission standardized to 15 °C (kg CH_4_ ha^–1^ year^–1^), *T*_15_ is the temperature used to standardize rates (where 15 °C is 288.15 K), *E*_M_ is the temperature sensitivity for methane emissions (eV t CH_4_ ha^–1^ year^–1^), and *k*_B_ is the Boltzmann constant (8.617 × 10^–5^ eV K^–1^). Malerba et al.^8^ calibrated this model using data from the scientific literature to estimate parameters *E*_M_ at 0.43 (95% C.I.: 0.21, 0.64) and ln[*M*_*i*_(*T*_15_)] at 204 (95% CI: 83–521). For *T*_*i*_, we extracted the mean monthly temperature for each agricultural pond using ANUClimate v2 (see Section 2.1.4).

We compared total methane emissions from the temperature-dependent (tier 3) method in eq 1 with two simpler methods. The first one is the temperature-independent (tier 1) method proposed by the IPCC, which applies a fixed emission factor of 0.183 t CH_4_ ha^–1^ year^–1^ (95% CI: 0.118–0.228) to all agricultural ponds, regardless of temperature or climate (Table 7.12 in Lovelock et al.^26^). The second one is the climate-dependent method (tier 2) used for the 2022 national GHG inventory of Australia, which assumes a constant emission factor for each climate in Australia: subtropical (0.381 t CH_4_ year^–1^ ha^–1^), temperate–cool (0.152), temperate–warm (0.238), tropical–dry (0.581), and tropical–wet (0.697).^27^ These climate-dependent coefficients follow the Köppen classification and were calculated using 56 observations (none from Australia) from four peer-reviewed articles. Conversely, the *ln*[*M*_*i*_(*T*_15_)] parameter of the temperature-dependent (tier 3) method was estimated by Malerba et al.^8^ using 286 observations (61% in Australia) from seven peer-reviewed articles.

### 2.3 Estimating Monthly Methane Emissions from Agricultural Ponds

We calculated total methane emissions from the 1.7 million agricultural ponds in Australia by multiplying the monthly time series of water surface area by the monthly methane emissions predicted using the temperature-corrected (tier 3) method. Specifically, we used the XGBoost model (Section 2.1.4) to calculate the monthly water surface from 1990 to 2022 for each pond (purple shapes in Figure 2) and the tier 3 model (eq 1 in Section 2.2) to predict the methane flux from the agricultural pond given the local temperature (red shapes in Figure 2). Aggregating the results for individual ponds over each financial year (i.e., from July first to June 30th—as required for national GHG inventories) and according to State and Territory provides the annual methane emissions reported in the inventory (green shapes in Figure 2).

Our methods estimate total methane emissions associated with agricultural ponds. In some national GHG inventories (e.g., Australia), the total methane emission from agricultural ponds is partitioned into baseline agricultural pond emissions (reported under the “Land Use, Land Use Change, and Forestry”) and emissions due to manure pollution (reported under “Manure Management” in “Agriculture”). The results in this paper report total methane emissions from agricultural ponds without applying this partitioning.

## 3 Results

### 3.1 Historical Increase in Agricultural Ponds

Australian agricultural ponds in 2022 (1.77 million) were 44% more than in 1990 (1.23 million; Table 1). More than half of these new ponds are in New South Wales and Victoria (Table 1). Yet, the regions with the highest rates of increase in the density of agricultural ponds are the Australian Capital Territory (121%) and Queensland (69%), followed by Tasmania (64%), Western Australia (52%), South Australia (51%), Victoria (37%), and New South Wales (36%; Table 1).

**Table 1:** Comparison of Density, Water Surface Area, and Total Methane Emissions of Agricultural Ponds in Australia and in Each State and Territories between 1990 and 2021^a^

	counts (thousands)				water surface area (kha)				emissions (kt CH_4_ year^–1^)				flux (t CH_4_ ha^–1^ year^–1^)			
region	year 1990	year 2022	rel. change	abs. diff	year 1990	year 2022	rel. change	abs. diff	year 1990	year 2022	rel. change	abs. diff	year 1990	year 2022	rel. change	abs. diff
NSW	480.5	655.7	36%	175.2	46.1	67.6	47%	21.6	10.27	15.5	51%	0.51	0.223	0.229	2.7%	0.006
VIC	322.2	441.2	37%	119.0	26.8	36.6	37%	9.8	5.29	7.45	41%	0.41	0.197	0.203	3.0%	0.006
QLD	140.3	237.0	69%	96.8	21.5	37.4	74%	15.9	6.22	11.42	84%	0.84	0.290	0.306	5.5%	0.016
WA	157.3	238.8	52%	81.5	11.9	17.1	44%	5.2	2.61	3.9	49%	0.49	0.219	0.228	4.1%	0.009
SA	77.7	117.5	51%	39.7	3.8	6.1	62%	2.3	0.78	1.29	65%	0.65	0.208	0.212	1.9%	0.004
TAS	38.8	63.8	64%	25.0	3.6	6.2	72%	2.6	0.62	1.09	76%	0.76	0.173	0.176	1.7%	0.003
NT	9.7	15.1	55%	5.4	0.9	1.5	61%	0.6	0.21	0.35	67%	0.67	0.228	0.236	3.5%	0.008
ACT	1.0	2.2	121%	1.2	0.2	0.4	133%	0.2	0.03	0.07	133%	1.33	0.200	0.200	0.0%	0
**AUS**	**1227**	**1771**	**44%**	**543.7**	**114.6**	**172.8**	**51%**	**58.2**	**26.03**	**41.07**	**58%**	**15.04**	**0.227**	**0.238**	**4.8%**	**0.011**

a Refer to Table S1 for yearly values for Australia. Due to a lack of historical data, the statistics for the Northern Territory are derived from the grand average across Australia. Australian regions are New South Wales (NSW), Victoria (VIC), Queensland (QLD), Western Australia (WA), South Australia (SA), Tasmania (TAS), Northern Territory (NT), and Australian Capital Territory (ACT). The national averages for Australia (AUS) are reported in bold. These predictions are reported in the 2024 national greenhouse gas inventory of Australia.

### 3.2 Water Surface Area of Agricultural Ponds

Manual tracing of surface area and theoretical maximum water surface area (including bare clay) using satellite images from 2009 to 2023 showed that Australian agricultural ponds across the year are on average at 62% capacity (median = 59%, first quantile = 38%, third quantile = 74%). Our model used to predict monthly fluctuations in the water surface of agricultural ponds explained much of this variability (Figure S2). Specifically, the correlation coefficient for the validation data set was 0.79 (95% CI: 0.73–0.83), and the mean absolute error (MAE) for predicting monthly time series of water surface was 0.065 ha (650 m^2^), which is equivalent to 46% mean absolute percentage error (MAPE; Figure S2). Also, the fitted line between observed and predicted water surfaces approached a 1:1 ratio and overlapped the line of equality (i.e., the dashed line overlapped the 95% confidence and prediction intervals in Figure S2).

**Figure 3:**
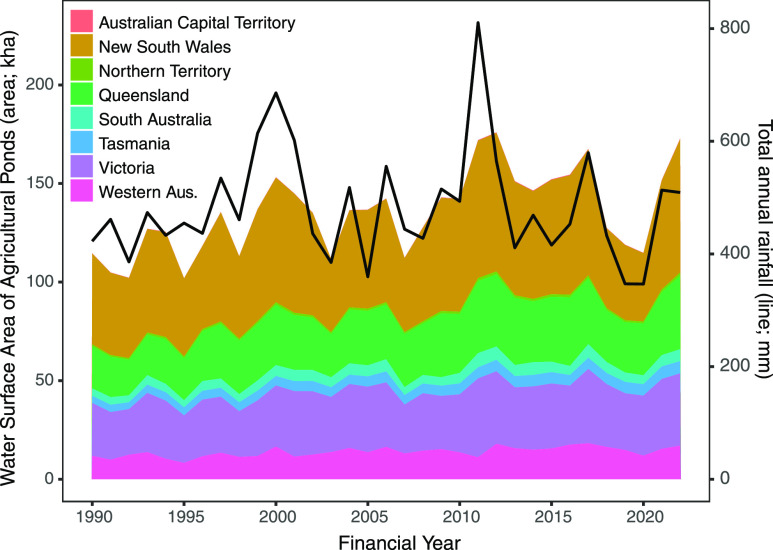
Total water surface of agricultural ponds divided by State and Territory and total annual rainfall in Australia. Changes in water surface are due to new ponds being established (see Figure S1) and climate conditions of rainfall and temperature affecting the relative water capacity (see Figure 4). Time is reported in financial years (1^st^ July to 30^th^ June), as required for the Australian National Inventory Report. See Tables 1 and S1 for raw data.

**Figure 4:**
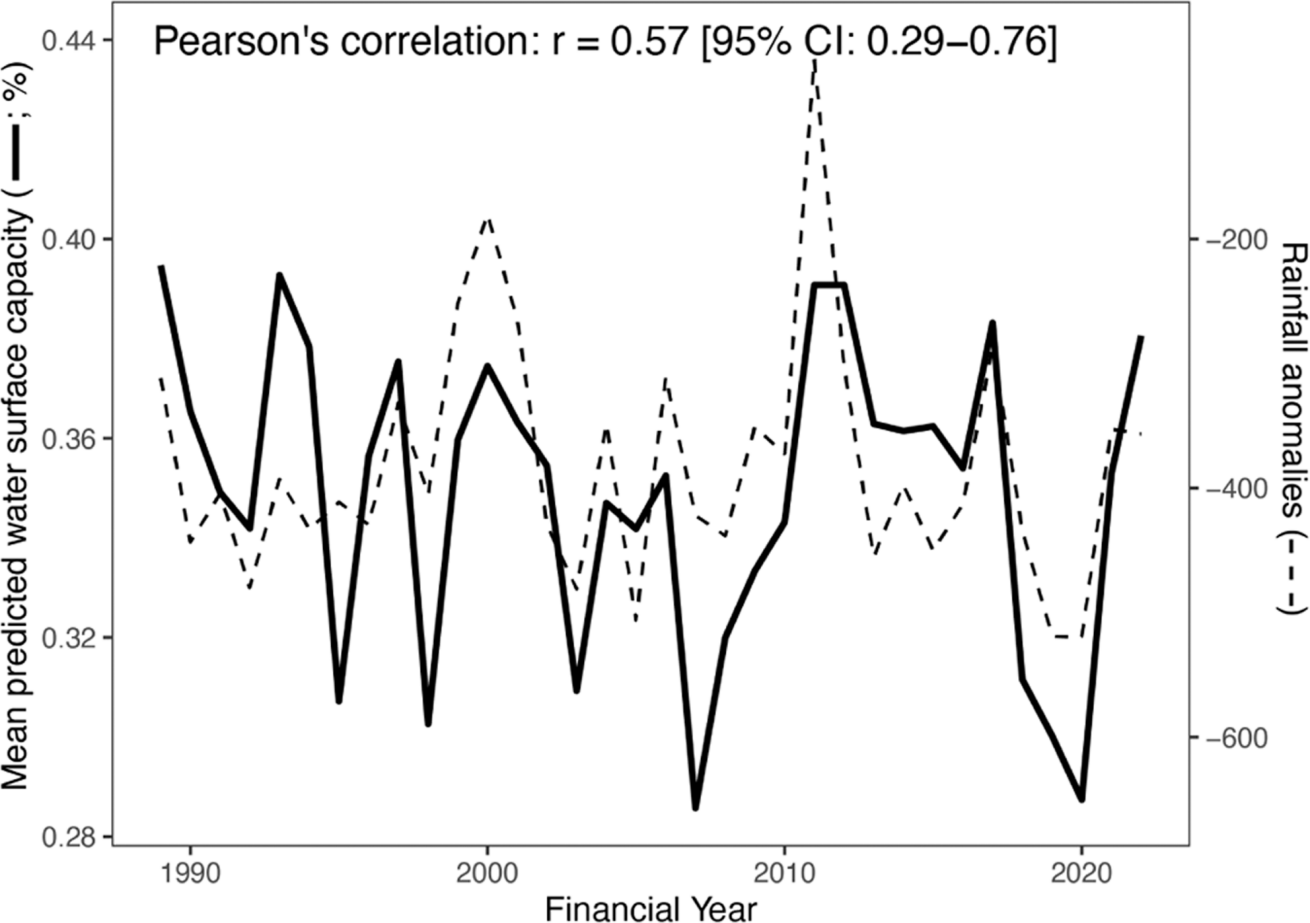
Australia-wide historical changes in the relative water capacity of agricultural ponds (solid line) and rainfall anomalies sourced from the Australian Bureau of Meteorology (dashed line). The correlation coefficient [±95% CI] between the relative water capacity and rainfall anomalies is reported on the plot (*r* = 0.57, *t* = 3.94, df = 32, *p* < 0.001). Time is reported in financial years (1^st^ July to 30^th^ June), as required for the Australian National Inventory Report.

Across Australia, the water surface of agricultural ponds increased by 51% between 1990 (115 kha, 1,150 km^2^) and 2022 (173 kha, 1,730 km^2^; Table 1 and Figure 3). The largest increases were in New South Wales (21.5 kha, 215 km^2^), Queensland (15.9 kha, 159 km^2^), and Victoria (9.8 kha, 98 km^2^; Table 1). Fluctuations in the total water surface of agricultural ponds were positively correlated with rainfall anomalies (*r* = 0.57, *t* = 3.94, df = 32, *p* < 0.001; Figures 3 and 4). These fluctuations correspond, on average, to an 11.5% change in the total water surface of agricultural ponds from 1 year to the next but up to 32% in some years (e.g., from 2020 to 2021; Figure 3).

### 3.3 Emission Factors (EFs) for Agricultural Ponds

**Figure 5:**
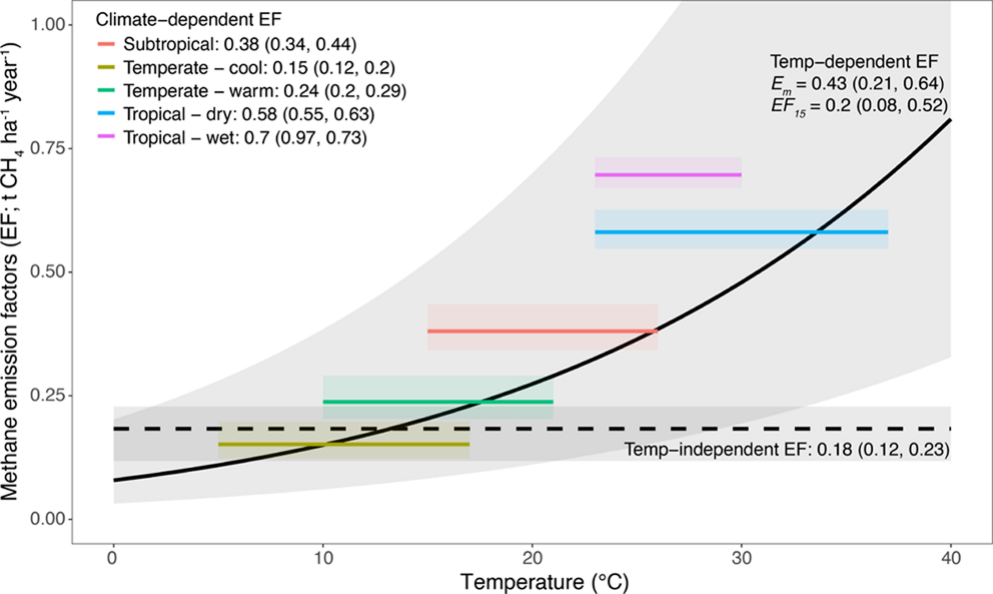
Comparing three methods to estimate the methane emission factor (or methane flux) of agricultural ponds in Australia (±95% CI). The first is the temperature-independent (tier 1) method proposed by the IPCC, which applies a fixed emission factor to all agricultural ponds (dashed line). The second is the climate-dependent method (tier 2) used for earlier editions of Australia's National GHG Inventory report, assuming a constant emission factor for each climate in Australia (solid, colored lines). The third is the temperature-dependent model (tier 3) developed in this study for the 2024 national GHG inventories of Australia using the Boltzmann–Arrhenius equation, which assumes a continuous exponential function of temperature (solid, black line). Emission factors for tiers 1 and 2 (units t CH_4_ ha^–1^ year^–1^), and temperature sensitivity parameters for activation energy (*E*_m_; eV) and emission factors at 15 °C (*E*_F15_; t CH_4_ ha^–1^ year^–1^) for tier 3 are reported in the figure (±95% CI).

Our temperature-dependent (tier 3) method to correct for temperature using the Boltzmann–Arrhenius relationship predicts an exponential increase of methane emissions from agricultural ponds with increasing temperatures. Specifically, an agricultural pond experiencing a mean annual temperature of 30 °C generates 3.2 times more emissions than one at 10 °C (0.48 vs 0.15 t ha^–1^ year^–1^; solid line in Figure 5).

Field data from Australian agricultural ponds confirmed a significant positive effect of temperature on methane emissions (*F*_1,175_ = 28.9, *p* < 0.001; Figure S3). Above 20 °C, mean emissions predicted by the model are consistent with the observed data (see overlapping blue and black lines in Figure S3). Yet, model predictions underestimate the increase in methane flux with increasing temperatures measured in Australian dams (notice the shallower slope of the black line compared to the blue one in Figure S3). Nonetheless, available data from the field show that the relationship between temperature and methane fluxes among agricultural ponds has a high unexplained variability (*R*^2^ = 0.14).

Compared to the temperature-dependent (tier 3) method used here, the temperature-independent EF (tier 1) method proposed by IPCC overpredicts emissions at temperatures cooler than 13 °C and underpredicts emissions at warmer temperatures. For example, emissions for sites with mean annual temperatures of 30 °C would be 2.7 times higher using the temperature-dependent model (0.48 t ha^–1^ year^–1^) compared to that of the temperature-independent model (0.18 t ha^–1^ year^–1^; compare solid and dashed lines in Figure 5).

Finally, the climate-specific EF (tier 2) used in previous editions of Australia’s national GHG inventory follows a similar increasing trend between methane emission and temperature as the temperature-dependent method (compare colored lines with a black solid line in Figure 5). Nonetheless, four of the five climate-specific coefficients were on average higher than those predicted with the temperature-dependent model (i.e., the midpoints of the colored lines are mostly above the black solid line in Figure 5). In particular, tropical wet, subtropical, tropical dry, and temperate warm climates were 75, 33, 18, and 11% higher, respectively, than the corresponding predictions from the temperature-dependent model based on the average temperatures of each climate (Figure 5).

### 3.4 Methane Flux and Total Emissions from Agricultural Ponds in Australia

**Figure 6:**
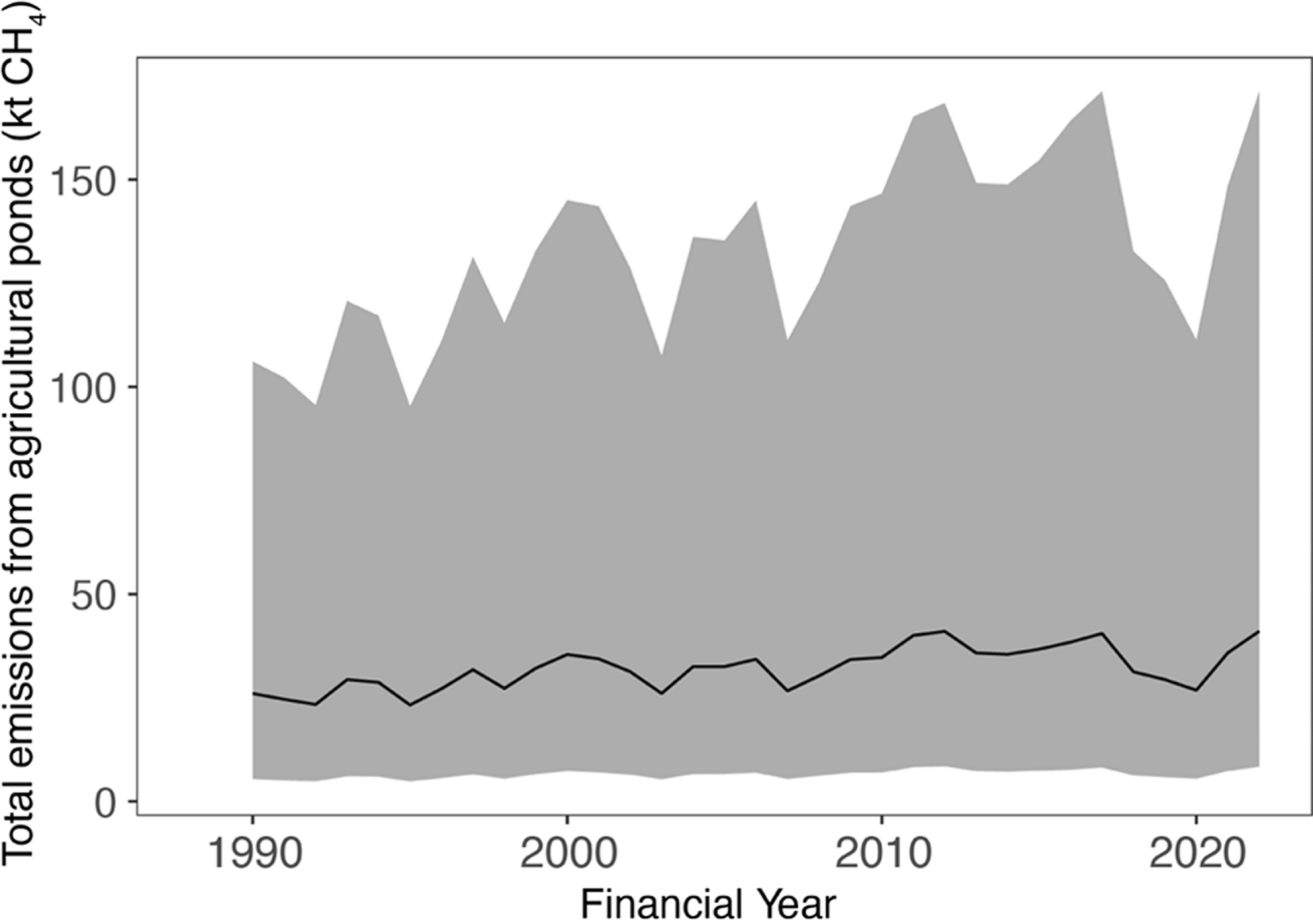
Total methane emissions (±95% C.I.) from agricultural ponds developed in this study for the 2024 national greenhouse gas inventory of Australia. Total emissions depend on the total predicted water surface area (see the area in Figure 3) and the methane flux calculated with the temperature-dependent model (see the solid line in Figure 5). The uncertainty of model predictions is associated with correcting for temperature, quantifying the total water surface area, and predicting methane emission factors (see Figure 7). Time is reported in financial years (1^st^ July to 30^th^ June), as required for the Australian National Inventory Report.

Our model predicts a 58% increase in total methane emissions from Australian agricultural ponds from 1990 (26 kt of CH_4_ year^–1^) to 2022 (41 kt of CH_4_ year^–1^; Figure 6 and Table 1, S1). On average, emissions increased by 0.34 kt year^–1^ with a 22% year-to-year variability (*F*_1,31_ = 21.26, *p* < 0.001). This increasing trend is explained by on average 16,000 agricultural ponds being established in Australia yearly since 1990, increasing the methane-emitting water surface area by 1.23 kha year^–1^. Also, model predictions show that the mean methane flux from agricultural ponds has increased by 3.5% from 1990 (0.230 t CH_4_ ha^–1^ year^–1^) to 2022 (0.238 t CH_4_ ha^–1^ year^–1^; *F*_1,31_ = 24.04, *p* < 0.001; Table 1, S1).

In 2022, the State with the highest methane emissions from agricultural ponds was New South Wales (15.5 kt year^–1^; Figure S4 and Table 1). Queensland recorded the second-highest emissions (6.2 kt year^–1^), followed by Victoria (5.3 kt year^–1^) and Western Australia (2.6 kt year^–1^; Figure S4 and Table 1).

### 3.5 Sensitivity Analysis and Parameter Uncertainty

**Figure 7:**
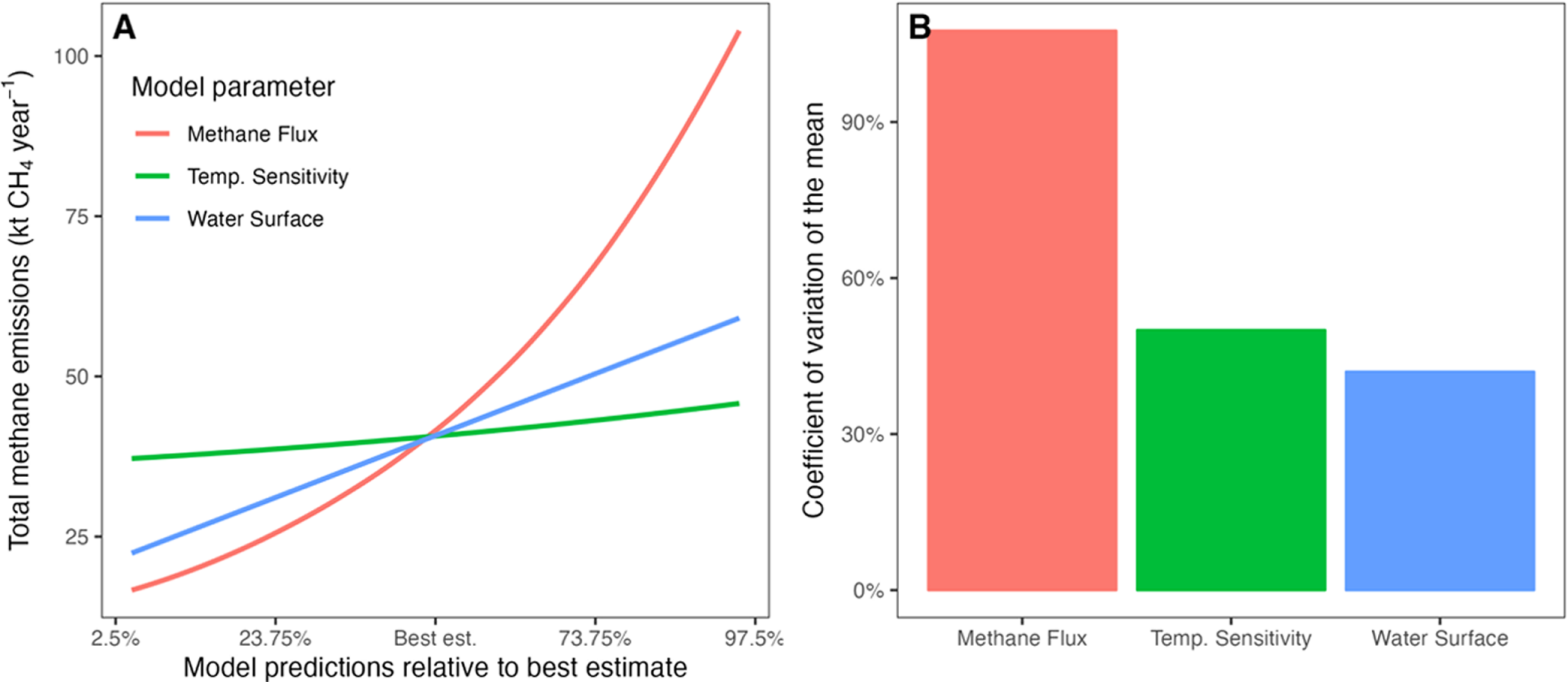
Sensitivity analysis and sources of uncertainty in the model. (A) Spider plot showing the sensitivity of model predictions across the 95% confidence interval of each parameter best estimate, with 2.5% as the lower bound and 97.5% as the upper bound. The parameters define the mean methane flux at 15 °C (parameter *M*_*i*_(*T*_15_) in eq 1), the temperature sensitivity (parameter *E*_*M*_ in eq 1), and the predictions of water surface area from the Extreme Gradient Boosting regression. (B) The coefficients of variation of the mean (i.e., standard error divided by the mean and multiplied by 100) are used to compare parameter uncertainties across model parameters.

The parameter with the highest repercussions on model predictions for the 2024 national GHG inventory (temperature-dependent EF) was the coefficient estimating the average methane flux of an agricultural pond at 15 °C (parameter *M*_*i*_(*T*_15_) in eq 1). This parameter has an exponential effect on model predictions, and increasing *M*_*i*_(*T*_15_) to the upper limit of the 95% confidence interval (from 0.204 to 0.521 t CH_4_ year^–1^ ha^–1^) would result in a 154% increase in the predicted total methane emissions from Australian agricultural ponds (from 41 to 104 kt CH_4_ year^–1^; Figure 7a). This parameter also exhibited the highest degree of uncertainty within the model, registering a coefficient of variation (CV) of 107% (Figure 7b).

The model was less sensitive to the uncertainty associated with predicting the monthly water surface of an agricultural pond (Figure 7). Our Extreme Gradient Boosting regression reported a CV of 42% (Figure 7b), and any changes in the water surface would produce a proportional effect on total methane emissions (Figure 7a).

Finally, the coefficient for the temperature sensitivity of methane emissions (parameter *E*_M_ in eq 1) exhibited the lowest sensitivity for model predictions. Specifically, increasing *M*_*i*_(*T*_15_) to the upper limit of the 95% confidence interval (from 0.43 to 0.64) produced a 12% increase in total emissions (from 41 to 46 kt CH_4_ year^–1^; Figure 7a). This parameter also recorded a CV of 50% (Figure 7b).

## 4 Discussion

Following the 2019 Refinement of IPCC guidelines, Nations are encouraged to account for methane emissions from small (<8 ha) constructed waterbodies in their National Greenhouse Gas Inventory Reports (hereafter “national GHG inventories”) as “Other Constructed Waterbodies” under “Land Use, Land Use Change, and Forestry”. This study presents the method to inform the 2024 edition of Australia’s national GHG inventory on methane emissions from small constructed waterbodies. Our method introduces several improvements by accounting for (1) monthly fluctuations in the surface area of individual ponds, (2) rates of historical accumulation of agricultural ponds, and (3) the temperature dependency of methane emissions in aquatic systems. Official data and reports for Australia’s national GHG inventories are available from UNFCCC and the Australian Dept. of Climate Change, Energy, the Environment, and Water (UN website; DCCEEW website).

**Figure 8:**
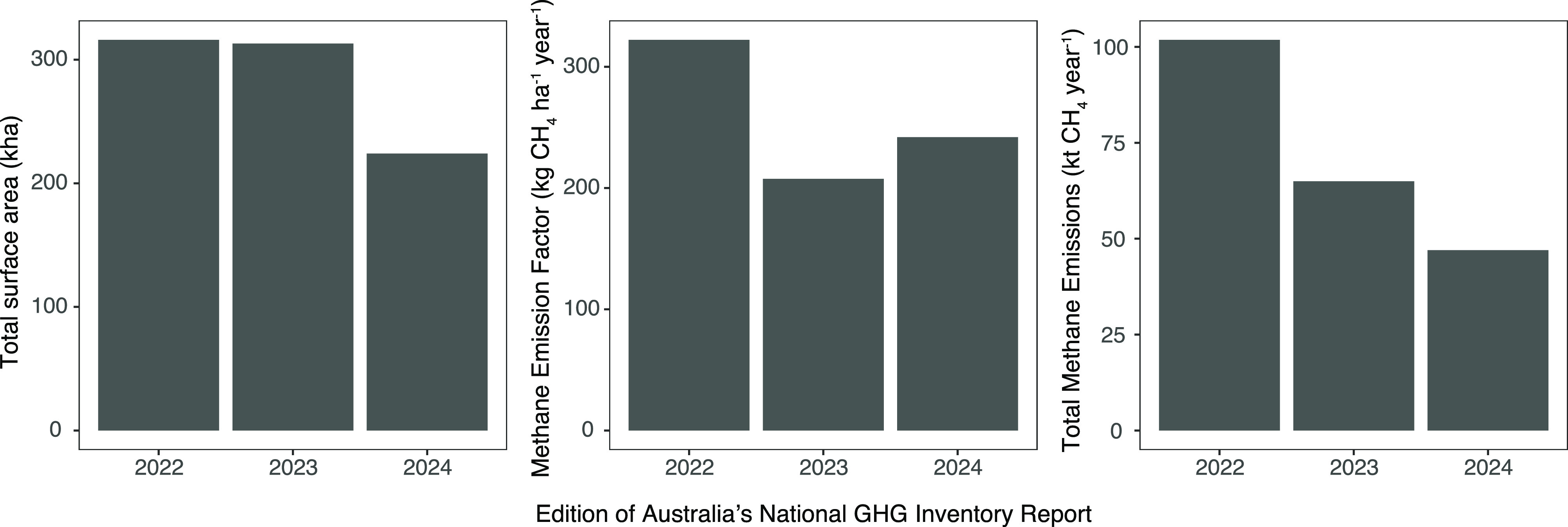
Annual national estimates for total water surface, methane emission factor (or methane flux), and total methane emissions of “Other Constructed Waterbodies” (most of which are agricultural ponds and reservoirs) as reported in the latest three editions of Australia’s National greenhouse gas inventory. This study informs the statistics for the 2024 edition. Note that the Australian national GHG inventory reports total methane emissions from constructed waterbodies divided into 60% “manure component” (reported under “Manure Management” in “Agriculture”) and 40% “baseline component” (reported under “Flooded land remaining flooded land” in “Land Use, Land Use Change, and Forestry”). Values in this plot are total emissions without this partitioning. See Table S1 for 95% confidence intervals for 2024 values of agricultural ponds.

Our model estimates Australian agricultural ponds to cover 173 kha (95% CI: 90 to 276) and emit 41 kt CH_4_ year^–1^ (95% CI: 8–194). Compared to previous editions of the national GHG inventory, agricultural ponds occupy a smaller water surface area (173 vs >300 kha), mainly because our model accounts for ponds partially covered by water (Figure 8). Specifically, our data show that agricultural ponds are on average at 62% capacity, indicating that previous predictions assuming full water capacity overestimate total methane emissions. Also, the methane flux predicted in our model for agricultural ponds using the Boltzmann–Arenious relationship is intermediate compared with previous editions of the national GHG inventory. The average annual methane flux (emissions per area) across Australia in our model for the 2024 edition (0.238 t CH_4_ ha^–1^ year^–1^) is 35% lower than the 2022 edition (0.322 t CH_4_ ha^–1^ year^–1^) and 14% higher than the 2023 edition (0.208 t CH_4_ ha^–1^ year^–1^) of Australia’s national GHG inventory (Figure 8). Finally, our methane flux for 2024 is 30% higher than the default fixed coefficient proposed in the 2019 IPCC guidelines for small constructed waterbodies (0.183 t CH_4_ ha^–1^ year^–1^). Overall, the 2024 edition of the Australian national GHG inventory predicts a lower total methane emission (47 t CH_4_ year^–1^) from all types of “Other Constructed Waterbodies” compared to the 2022 (102 kt CH_4_ year^–1^) and 2023 (65 kt CH_4_ year^–1^) editions (Figure 8).

National GHG inventories serve as a country’s official record for tracking emissions over time and assessing progress toward international climate agreements, such as the Kyoto Protocol or the Paris Agreement.^28,29^ A common objective of international climate efforts is to reduce emissions to levels observed in a baseline year, which is frequently 1990.^29^ Accurately representing historical changes in anthropogenic emissions is crucial for defining emission reduction targets to revert to 1990 levels. Our approach accounts for a 44% increase in pond surface area (from 115 to 173 kha) and a 58% increase in total emissions (from 26 to 41 kt CH_4_ year^–1^) since 1990. Conversely, the 2022 edition of the national GHG inventory estimated a 15% increase in surface area (from 275 to 316 kha) and a 20% increase in emissions (from 84 to 102 kt CH_4_ year^–1^) since 1990. Finally, the 2023 edition of the national GHG inventory estimated a 34% increase in surface area (from 233 to 313 kha) and a 242% increase in emissions (from 19 to 65 kt CH_4_ year^–1^) since 1990. Overall, the emission reduction targets for agricultural ponds to return to 1990 levels in the 2024 edition of Australia’s national GHG inventory (15 kt CH_4_ year^–1^) are comparable to the 2023 edition (18 kt CH_4_ year^–1^) and are lower than the 2022 edition (46 kt CH_4_ year^–1^).

In addition to small artificial ponds, established (>10 years old) reservoirs represent the other major category of artificial waterbodies identified in national GHG inventories to report methane emissions.^17^ As for agricultural ponds, reservoirs are also the focus of research to estimate their contributions to anthropogenic methane emissions more accurately.^30^ Compared to reported statistics for established reservoirs in the 2024 national GHG inventory for the year 2022, agricultural ponds occupy 56% less surface area (173 vs 394 kha), yet they emit twice as much methane per area (0.238 vs 0.119 t CH_4_ ha^–1^) and similar methane in total (41 vs 47 kt CH_4_ year^–1^). However, emissions from established reservoirs have increased by 147% from 1990 to 2022 (from 19 to 47 kt CH_4_ year^–1^), against the 58% increase (from 26 to 41 kt CH_4_ year^–1^) from agricultural ponds. Finally, there are negligible emissions from reservoirs that are less than 10 years old (0.51 kt CH_4_ year^–1^). Overall, methane emissions from all constructed waterbodies (most of which are agricultural ponds and reservoirs) reported in Australia’s 2024 national GHG inventory report for 2022 are 94 kt CH_4_ year^–1^. This amount represents 18% of all anthropogenic CH_4_ emissions from the land use, land use change, and forestry sector (499 kt CH_4_ year^–1^), 4.2% from the agricultural sector (2,209 kt CH_4_ year^–1^), and 2.1% from all sectors (4,373 kt CH_4_ year^–1^) reported for 2022.

The uncertainty detected in our model informs future research priorities. In particular, emissions per area from an average agricultural pond at 15 °C had the highest uncertainty in the model, with a mean of 0.204 t of CH_4_ ha^–1^ year^–1^ and confidence intervals ranging from 0.083 to 0.521 t of CH_4_ ha^–1^ year^–1^. Part of this 6-fold difference is due to variability from pond management, which is unaccounted for in our model. For example, fencing agricultural ponds to exclude livestock from entering the water can improve water quality and halve aquatic methane emissions by reducing the direct deposition of nutrient-rich manure and urine into the water.^31^ Also, the type of agricultural pond influences aquatic emissions, with livestock ponds emitting up to twice as much as cropping and urban ponds.^6,7^ Moreover, smaller ponds typically have higher methane emissions per area than larger ones, likely because of higher nutrient concentrations from a larger perimeter-to-surface ratio.^32^ Future research should develop remote sensing tools to quantify important features of individual ponds and better predict their emissions. Yet, developing context-specific emission factors (tier 3) requires more field measurements. For example, most studies only focus on diffusive methane fluxes and omit ebullitive ones, which in warm climates make up most of the total methane emissions of a pond.^7,8^

Another major step forward to improve nationwide methane emissions from agricultural ponds is to improve the parameter for the temperature sensitivity of methanogenesis. Our model for the 2024 Australian national GHG inventory uses the Boltzmann–Arrhenius relationship to account for the effects of temperature on the emission factor of aquatic systems, which differs from previous attempts using either a temperature-independent factor (e.g., IPCC guidelines) or climate-specific emission factors (e.g., the 2022 Australian national GHG inventory). Our approach captures variability at smaller geographical scales, does not require arbitrary decisions on the definition of each climate, and avoids the artifact of large differences in emissions for nearby ponds at the edge of climate zones. However, the temperature sensitivity coefficient (parameter *E*_m_) in the Boltzmann–Arrhenius relationship reveals wide confidence intervals (from 0.2 to 0.6 eV) due to variability in field data. Also, in the absence of data, *E*_m_ was estimated using larger systems, such as lakes and reservoirs, as described in Malerba et al.^8^ This approach is likely conservative for agricultural ponds. Freshwater systems of comparable sizes to agricultural ponds often record values around 1 eV,^33,34^ whereas larger freshwater systems have lower values around 0.4–0.6.^35,36^ Increasing the temperature sensitivity of our model would increase the total emissions. For example, increasing *E*_m_ from 0.4 to 1 eV would increase our predicted methane emissions from agricultural ponds by 44% (from 41 to 59 kt CH_4_ year^–1^).

All data, models, and statistics for replicating our methodology are accessible via the free cloud-based server DEA Sandbox (see Methods S1 for detailed instructions). The model is executed in Python and organized in six Jupyter Notebooks. The codes available in the DEA Sandbox can be executed to replicate our analyses for Australia. Extending similar statistics for other countries would require the construction of training data consisting of agricultural pond sizes and water areas along with the associated monthly climate rainfall and temperature variables taken from other countries. Our code could then be used to train new XGBoost models tailored for the particular geographical area. Additional data would also be required to use these models, including the size of every agricultural pond and climate variable for the particular geographical extent and temporal range required. However, there is uncertainty about the training data set required to generate useful predictions on the water surface and methane emissions of agricultural ponds worldwide.

By improving our understanding of greenhouse gas emissions from small agricultural ponds, we hope that our research can help promote innovative, scalable, and cost-effective mitigation strategies. Carbon credits are a compelling catalyst for channeling investments into low-carbon technologies. The global carbon credit market has surged impressively, boasting a 33% annual growth rate, from USD 85 billion in 2020 to USD 142 billion in 2022.^37^ This upward trajectory may continue as more countries and corporations embrace carbon pricing, possibly extending it to artificial freshwater systems. For example, increasing vegetation around agricultural ponds can improve water quality,^31,38^ increase livestock health,^39^ reduce emissions,^31^ and offer breeding habitat for local wildlife.^40^ Using nature-based solutions to reduce emissions while improving biodiversity in agricultural ponds may also attract funding from biodiversity finance, estimated at USD 78–91 billion.^41^ Together, these financial schemes can mobilize broad investments to increase the ecological and environmental value of agricultural ponds and boost farm productivity.

## Data Availability

All necessary data, models, and statistics for replicating our methods are accessible via the free cloud-based server DEA Sandbox (refer to Method S1 for detailed instructions). Most data are also available through an online interactive platform at AusDams.org, which allows the user to navigate to any area of Australia to generate tailored statistics, plots, and tables on various aspects of farm dams. All codes in Python to reproduce our analyses can be found in a free cloud-based platform (see Method S1 for instructions) and in a public repository at https://github.com/DPIRD-DMA/Weather-to-water/tree/master.

## Supporting Information Available

Figure S1: Observed and projected increases in pond numbers from 1989 to 2020; Figure S2: validation of the Extreme Gradient Boosting model; Figure S3: temperature dependency of the methane emission factor; Figure S4: total methane emissions from agricultural ponds; Table S1: yearly summary of agricultural ponds in Australia; and Method S1: methods to run the models presented in the study (PDF)

## References

[ref1] Masson-Delmotte V. Climate Change 2021: The Physical Science Basis: Working Group I Contribution to the Sixth Assessment Report of the Intergovernmental Panel on Climate Change; Cambridge University Press, 2021.

[ref2] Dlugokencky E. J.; Nisbet E. G.; Fisher R.; Lowry D. Global Atmospheric Methane: Budget, Changes and Dangers. Philos. Trans. R. Soc., A 2011, 369 (1943), 2058–2072. 10.1098/rsta.2010.0341. 21502176

[ref3] Saunois M.; Stavert A. R.; Poulter B.; Bousquet P.; Canadell J. G.; Jackson R. B.; Raymond P. A.; Dlugokencky E. J.; Houweling S.; Patra P. K.; Ciais P.; Arora V. K.; Bastviken D.; Bergamaschi P.; Blake D. R.; Brailsford G.; Bruhwiler L.; Carlson K. M.; Carrol M.; Castaldi S.; Chandra N.; Crevoisier C.; Crill P. M.; Covey K.; Curry C. L.; Etiope G.; Frankenberg C.; Gedney N.; Hegglin M. I.; Höglund-Isaksson L.; Hugelius G.; Ishizawa M.; Ito A.; Janssens-Maenhout G.; Jensen K. M.; Joos F.; Kleinen T.; Krummel P. B.; Langenfelds R. L.; Laruelle G. G.; Liu L.; Machida T.; Maksyutov S.; McDonald K. C.; McNorton J.; Miller P. A.; Melton J. R.; Morino I.; Müller J.; Murguia-Flores F.; Naik V.; Niwa Y.; Noce S.; O’Doherty S.; Parker R. J.; Peng C.; Peng S.; Peters G. P.; Prigent C.; Prinn R.; Ramonet M.; Regnier P.; Riley W. J.; Rosentreter J. A.; Segers A.; Simpson I. J.; Shi H.; Smith S. J.; Steele L. P.; Thornton B. F.; Tian H.; Tohjima Y.; Tubiello F. N.; Tsuruta A.; Viovy N.; Voulgarakis A.; Weber T. S.; van Weele M.; van der Werf G. R.; Weiss R. F.; Worthy D.; Wunch D.; Yin Y.; Yoshida Y.; Zhang W.; Zhang Z.; Zhao Y.; Zheng B.; Zhu Q.; Zhu Q.; Zhuang Q. The Global Methane Budget 2000–2017. Earth Syst. Sci. Data 2020, 12 (3), 1561–1623. 10.5194/essd-12-1561-2020.

[ref4] Rosentreter J. A.; Borges A. V.; Deemer B. R.; Holgerson M. A.; Liu S.; Song C.; Melack J.; Raymond P. A.; Duarte C. M.; Allen G. H.; Olefeldt D.; Poulter B.; Battin T. I.; Eyre B. D. Half of Global Methane Emissions Come from Highly Variable Aquatic Ecosystem Sources. Nat. Geosci. 2021, 14 (4), 225–230. 10.1038/s41561-021-00715-2.

[ref5] Downing J. A.; Prairie Y. T.; Cole J. J.; Duarte C. M.; Tranvik L. J.; Striegl R. G.; McDowell W. H.; Kortelainen P.; Caraco N. F.; Melack J. M.; Middelburg J. J. The Global Abundance and Size Distribution of Lakes, Ponds, and Impoundments. Limnol. Oceanogr. 2006, 51 (5), 2388–2397. 10.4319/lo.2006.51.5.2388.

[ref6] Ollivier Q. R.; Maher D. T.; Pitfield C.; Macreadie P. I. Punching above Their Weight: Large Release of Greenhouse Gases from Small Agricultural Dams. Global Change Biol. 2019, 25 (2), 721–732. 10.1111/gcb.14477. 30457192

[ref7] Grinham A.; Albert S.; Deering N.; Dunbabin M.; Bastviken D.; Sherman B.; Lovelock C. E.; Evans C. D. The Importance of Small Artificial Water Bodies as Sources of Methane Emissions in Queensland, Australia. Hydrol. Earth Syst. Sci. 2018, 22 (10), 5281–5298. 10.5194/hess-22-5281-2018.

[ref8] Malerba M. E.; de Kluyver T.; Wright N.; Schuster L.; Macreadie P. I. Methane Emissions from Agricultural Ponds Are Underestimated in National Greenhouse Gas Inventories. Commun. Earth Environ. 2022, 3 (1), 1–7. 10.1038/s43247-022-00638-9.

[ref9] UNFCCC. United Nations Framework Convention on Climate Change 1992 Https://unfccc.int/resource/docs/convkp/conveng.pdf (accessed on April 10, 2024).

[ref10] Eggleston H. S.; Buendia L.; Miwa K.; Ngara T.; Tanabe K. IPCC Guidelines for National Greenhouse Gas Inventories 2006 2006 https://www.osti.gov/etdeweb/biblio/20880391 (accessed on April 10, 2024).

[ref11] IPCC. 2019 Refinement to the 2006 IPCC Guidelines for National Greenhouse Gas Inventories. 2019.

[ref12] Swartz T. M.; Miller J. R. The American Pond Belt: An Untold Story of Conservation Challenges and Opportunities. Front. Ecol. Environ. 2021, 19 (9), 501–509. 10.1002/fee.2381.

[ref13] Malerba M. E.; Wright N.; Macreadie P. I. Australian Farm Dams Are Becoming Less Reliable Water Sources under Climate Change. Sci. Total Environ. 2022, 829, 154360 10.1016/j.scitotenv.2022.154360. 35283121

[ref14] Tang J.; Zhuang Q.; Shannon R. D.; White J. R. Quantifying Wetland Methane Emissions with Process-Based Models of Different Complexities. Biogeosciences 2010, 7 (11), 3817–3837. 10.5194/bg-7-3817-2010.

[ref15] Bastviken D.; Cole J.; Pace M.; Tranvik L. Methane Emissions from Lakes: Dependence of Lake Characteristics, Two Regional Assessments, and a Global Estimate. Global Biogeochem. Cycles 2004, 18 (4), GB4009 10.1029/2004GB002238.

[ref16] Malerba M. E.; Wright N.; Macreadie P. I. A Continental-Scale Assessment of Density, Size, Distribution and Historical Trends of Farm Dams Using Deep Learning Convolutional Neural Networks. Remote Sens. 2021, 13 (2), 319 10.3390/rs13020319.

[ref17] Australian Government. In Quarterly Update of Australia’s National Greenhouse Gas Inventory: March 2023; Department of Climate Change, Energy, the Environment and Water, 2023.

[ref18] Mueller N.; Lewis A.; Roberts D.; Ring S.; Melrose R.; Sixsmith J.; Lymburner L.; McIntyre A.; Tan P.; Curnow S.; Ip A. Water Observations from Space: Mapping Surface Water from 25years of Landsat Imagery across Australia. Remote Sens. Environ. 2016, 174, 341–352. 10.1016/j.rse.2015.11.003.

[ref19] Krause C. E.; Newey V.; Alger M. J.; Lymburner L. Mapping and Monitoring the Multi-Decadal Dynamics of Australia’s Open Waterbodies Using Landsat. Remote Sens. 2021, 13 (8), 1437 10.3390/rs13081437.

[ref20] Mueller N.; Lewis A.; Roberts D.; Ring S.; Melrose R.; Sixsmith J.; Lymburner L.; McIntyre A.; Tan P.; Curnow S. Water Observations from Space: Mapping Surface Water from 25 Years of Landsat Imagery across Australia. Remote Sens. Environ. 2016, 174, 341–352. 10.1016/j.rse.2015.11.003.

[ref21] Chen T.; Guestrin C. XGBoost: A Scalable Tree Boosting System, Proceedings of the 22nd ACM SIGKDD International Conference on Knowledge Discovery and Data Mining; KDD ’16; Association for Computing Machinery: New York, NY, USA, 2016; pp 785–794.

[ref22] Hutchinson M. F.; Xu T. Anusplin Version 4.2 User Guide. Centre for Resource and Environmental Studies; The Australian National University: Canberra, 2004, 54.

[ref23] Hutchinson M. F.; Xu T.; Kesteven J. L.; Marang I. J.; Evans B. J. ANUClimate v2.0 (dataset), NCI Australia 2021 10.25914/60a10aa56dd1b (accessed on April 10, 2024).

[ref24] Hutchinson M. F.; Xu T.; Fenner School of Environment and Society. ANUSPLIN Version 4.4, Australian National University 2013 Https://fennerschool.anu.edu.au/research/products/anusplin (accessed on April 10, 2024).

[ref25] Nogueira F. Bayesian Optimization: Open Source Constrained Global Optimization Tool for Python 2014 https://github.com/fmfn/BayesianOptimization (accessed on April 10, 2024).

[ref26] Lovelock C. E.; Evans C.; Barros N.; Prairie Y.; Alm J.; Bastviken D.; Beaulieu J. J.; Garneau M.; Harby A.; Harrison J.; Pare D.; Raadal H. L.; Sherman B.; Zhang C.; Ogle S. M. 2019 Refinement to the 2006 IPCC Guidelines for National Greenhouse Gas Inventories; Zhongming Z.; Linong L.; Xiaona Y.; Wangqiang Z.; Wei L., Eds.; Wetlands, 2019; Vol. 4, p 7.

[ref27] National Inventory Report. Australian Government Dept. of Industry Science Energy and Resources. https://unfccc.int/documents/478957 (accessed on April 10, 2024).

[ref28] Breidenich C.; Magraw D.; Rowley A.; Rubin J. W. The Kyoto Protocol to the United Nations Framework Convention on Climate Change. Am. J. Int. Law 1998, 92 (2), 315–331. 10.2307/2998044.

[ref29] Seo S. N. Beyond the Paris Agreement: Climate Change Policy Negotiations and Future Directions. Reg. Sci. Policy Pract. 2017, 9 (2), 121–140. 10.1111/rsp3.12090.

[ref30] Delwiche K. B.; Harrison J. A.; Maasakkers J. D.; Sulprizio M. P.; Worden J.; Jacob D. J.; Sunderland E. M. Estimating Drivers and Pathways for Hydroelectric Reservoir Methane Emissions Using a New Mechanistic Model. J. Geophys. Res. Biogeosci. 2022, 127 (8), e2022JG006908 10.1029/2022JG006908.

[ref31] Malerba M. E.; Lindenmayer D. B.; Scheele B. C.; Waryszak P.; Yilmaz I. N.; Schuster L.; Macreadie P. I. Fencing Farm Dams to Exclude Livestock Halves Methane Emissions and Improves Water Quality. Global Change Biol. 2022, 28 (15), 4701–4712. 10.1111/gcb.16237. PMC9327511 35562855

[ref32] Holgerson M. A.; Raymond P. A. Large Contribution to Inland Water CO2 and CH4 Emissions from Very Small Ponds. Nat. Geosci. 2016, 9 (3), 222–226. 10.1038/ngeo2654.

[ref33] Chen H.; Xu X.; Fang C.; Li B.; Nie M. Differences in the Temperature Dependence of Wetland CO2 and CH4 Emissions Vary with Water Table Depth. Nat. Clim. Change 2021, 11 (9), 766–771. 10.1038/s41558-021-01108-4.

[ref34] Yvon-Durocher G.; Allen A. P.; Bastviken D.; Conrad R.; Gudasz C.; St-Pierre A.; Thanh-Duc N.; del Giorgio P. A. Methane Fluxes Show Consistent Temperature Dependence across Microbial to Ecosystem Scales. Nature 2014, 507 (7493), 488–491. 10.1038/nature13164. 24670769

[ref35] Li J.; Pei J.; Fang C.; Li B.; Nie M. Opposing Seasonal Temperature Dependencies of CO2 and CH4 Emissions from Wetlands. Global Change Biol. 2023, 29 (4), 1133–1143. 10.1111/gcb.16528. 36385719

[ref36] Kraemer B. M.; Chandra S.; Dell A. I.; Dix M.; Kuusisto E.; Livingstone D. M.; Schladow S. G.; Silow E.; Sitoki L. M.; Tamatamah R.; McIntyre P. B. Global Patterns in Lake Ecosystem Responses to Warming Based on the Temperature Dependence of Metabolism. Global Change Biol. 2017, 23 (5), 1881–1890. 10.1111/gcb.13459. 27591144

[ref37] World Bank. State and Trends of Carbon Pricing 2022; World Bank, 2022.

[ref38] Westgate M. J.; Crane C.; Smith D.; O’Malley C.; Siegrist A.; Florance D.; Lang E.; Crane M.; Hingee K.; Scheele B. C.; Lindenmayer D. B. Improved Management of Farm Dams Increases Vegetation Cover, Water Quality, and Macroinvertebrate Biodiversity. Ecol. Evol. 2022, 12 (3), e8636 10.1002/ece3.8636. 35342565 PMC8928867

[ref39] Dobes L.; Crane M.; Higgins T.; Van Dijk A.; Lindenmayer D. B. Increased Livestock Weight Gain from Improved Water Quality in Farm Dams: A Cost-Benefit Analysis. PLoS One 2021, 16 (8), e0256089 10.1371/journal.pone.0256089. 34398923 PMC8366965

[ref40] Boissinot A.; Besnard A.; Lourdais O. Amphibian Diversity in Farmlands: Combined Influences of Breeding-Site and Landscape Attributes in Western France. Agric. Ecosyst. Environ. 2019, 269, 51–61. 10.1016/j.agee.2018.09.016.

[ref41] OECD. A Comprehensive Overview of Global Biodiversity FinanceOrganisation for Economic Cooperation and Development (OECD) 2020 https://www.oecd.org/environment/resources/biodiversity/report-a-comprehensive-overview-of-global-biodiversity-finance.pdf (accessed on April 10, 2024).

