# Supplementary material for: Including Methane Emissions from Agricultural Ponds in National Greenhouse Gas Inventories
Source: Environ Sci Technol. 2024 May 2;58(19):8349–59. doi: 10.1021/acs.est.3c08898 (PMC11097397; doi:10.1021/acs.est.3c08898)
Supplement: Supplementary file 1 — es3c08898_si_001.pdf [file es3c08898_si_001.pdf]

Title:

Including methane emissions from agricultural ponds in national greenhouse gas inventories.

Martino E. Malerba [1,*], Tertius de Kluyver [2], Nicholas Wright [3], Omosalewa Odebiri [1], and Peter I. Macreadie [1].

[1] Deakin Marine Research and Innovation Centre, School of Life and Environmental Sciences, Deakin University, Melbourne, VIC 3125, Australia

[2] Australian Department of Climate Change, Energy, the Environment and Water; Emissions Reduction Division, Canberra, ACT, Australia.

[3] Sustainability and Biosecurity, Department of Primary Industries and Regional Development, 1 Nash St, Perth WA 6000, Australia.

* Corresponding author: m.malerba@deakin.edu.au; ORCID ID: https://orcid.org/0000-0002-7480-4779

Summary: 9 pages, 4 figures, 1 table, 1 method.

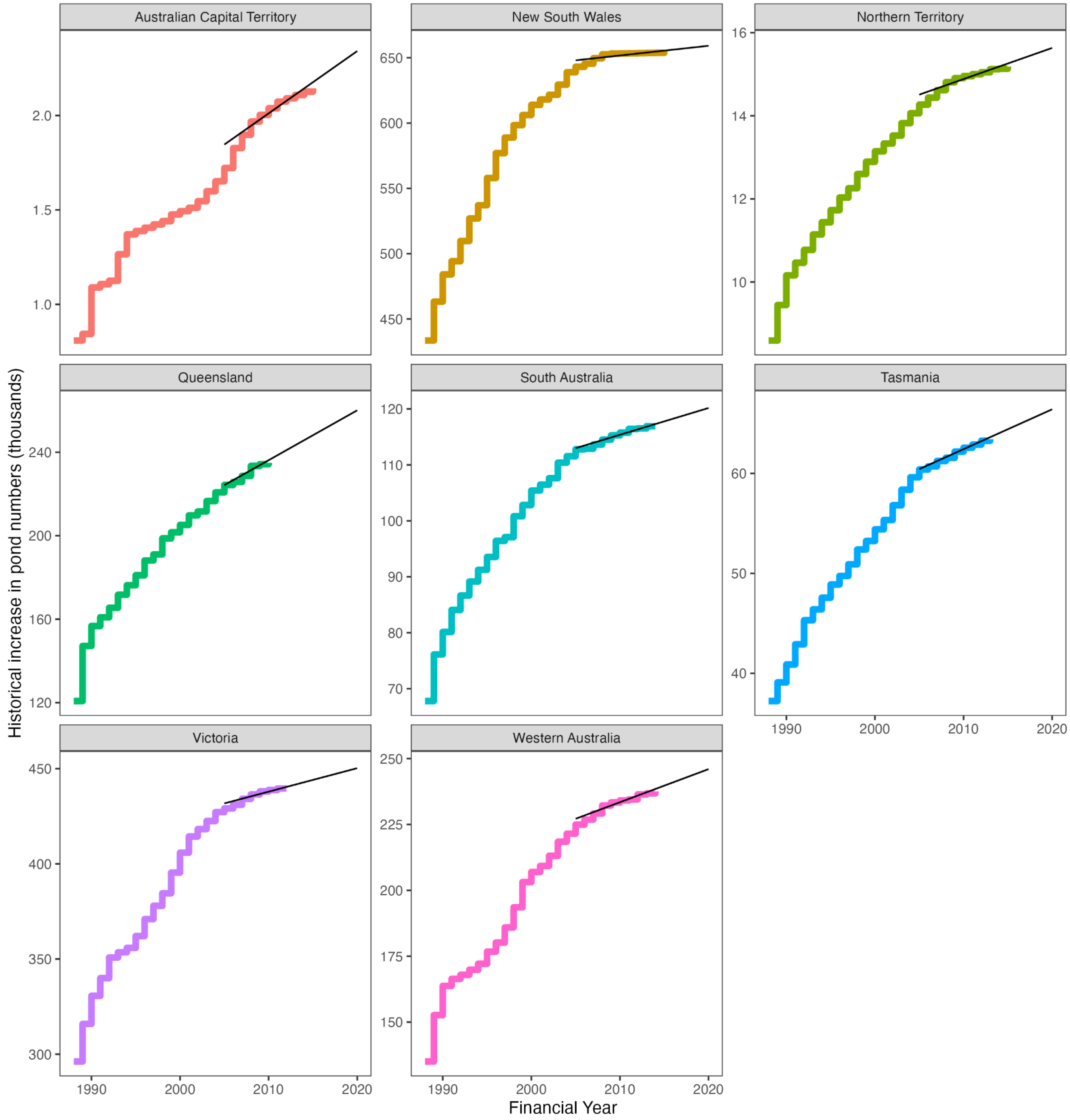

Figure S1: Observed (coloured lines) and projected (black lines) increases in pond numbers from 1989 to 2020. Projections from 2015 to 2020 use the average annual rates calculated between 2010 and 2015 for each State and Territory. Because there were too few documented dams in the Northern Territory to extract a reliable rate of increase, we used Australia's average rate of pond increase together with the estimated total pond density for this region in 2021. Time is reported in financial years (1st July to 30th June), as required for the Australian National Inventory Report.

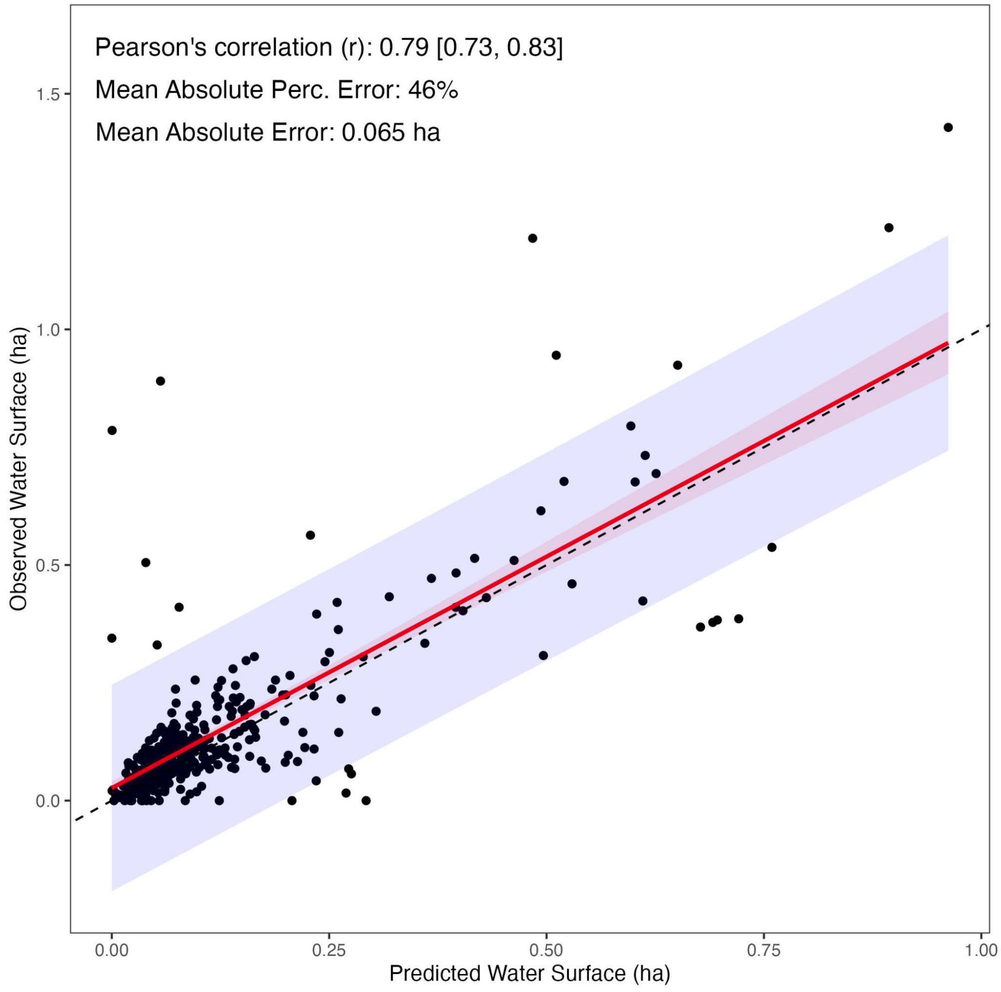

Figure S2: Validation of the Extreme Gradient Boosting model to estimate the surface area of an agricultural pond based on local weather and pond characteristics. Each point indicates the model predicted and the manually traced water surface area of an agricultural pond in Australia (N = 381). The dashed line shows where predictions match observations (i.e., identity line). The solid red line shows the line of best fit (±95% confidence intervals). The blue shading indicates the 95% prediction intervals. Indicated in the plot are the Pearson's correlation (r) [±95% confidence intervals], mean absolute percent error (MAPE), and mean absolute error (MAE).

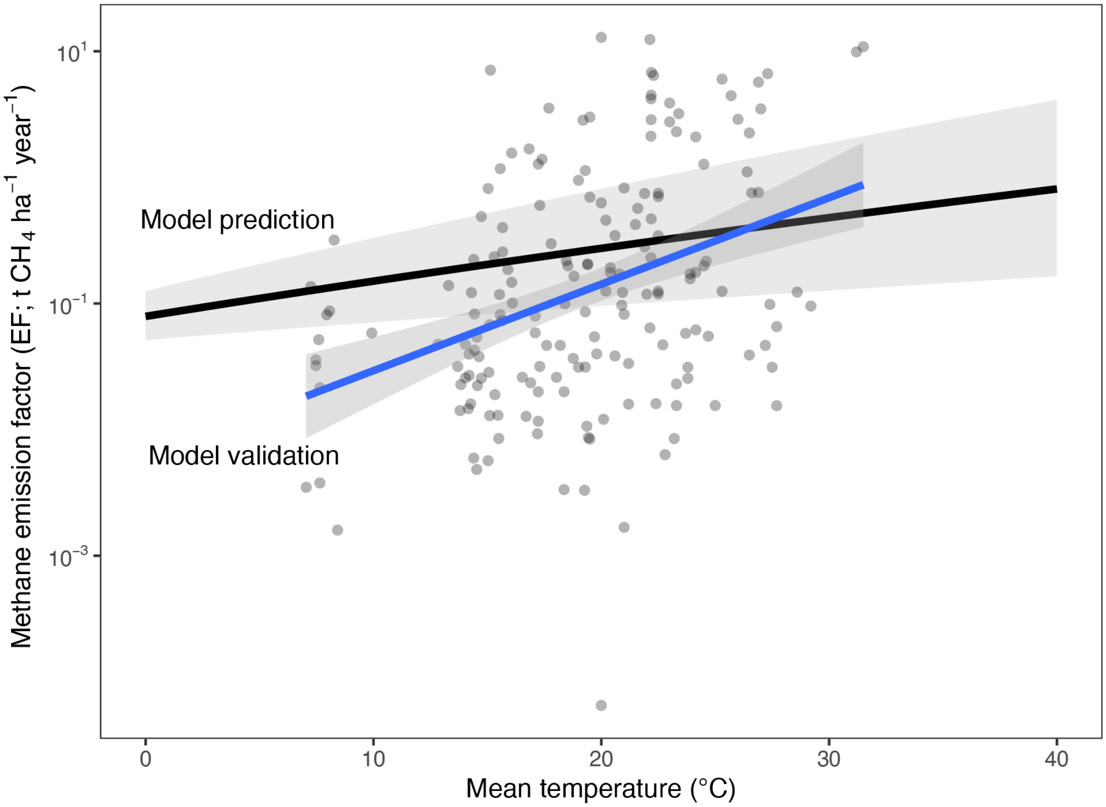

Figure S3: Temperature dependency of the methane emission factor (or methane flux) of agricultural ponds. Points are data from Australian ponds reported in the scientific literature. The black line shows model predictions using the temperature-corrected (Tier 3) method (see solid line in Figure 5). The blue line is the model validation, as the best-fitting linear model on the data ($R^2 = 0.14$, $F_{1,175} = 28.9$, $p < 0.001$).

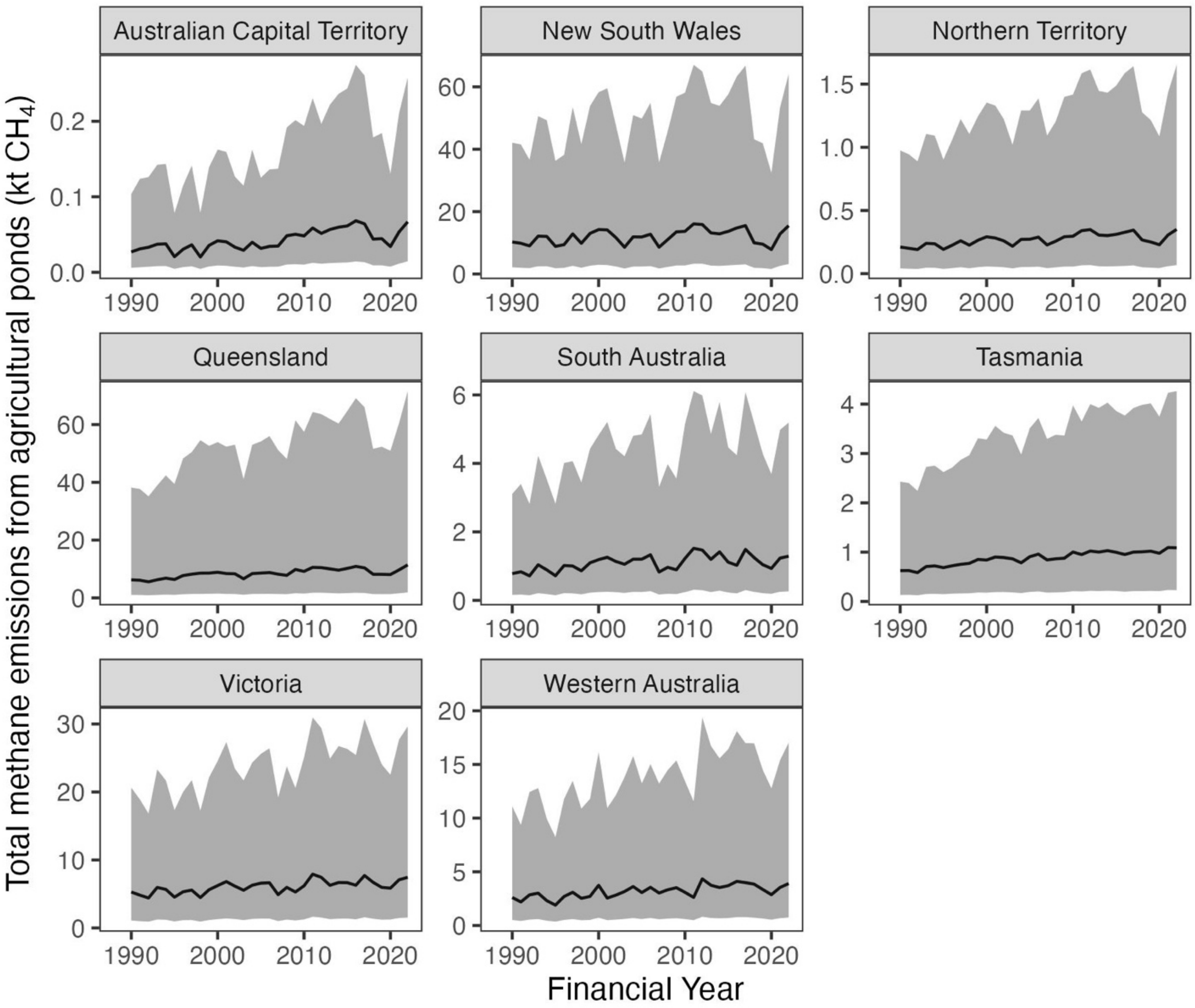

Figure S4: Total methane emissions from agricultural ponds in each State and Territory (±95% C.I.) of Australia developed in this study for the 2024 national greenhouse gas inventory of Australia. Total emissions depend on the total predicted water surface area (see Figure 3) and the methane flux calculated with the temperature-dependent model (see solid line in Figure 5). Refer to Figure 6 for nation-wide predictions. The uncertainty of model predictions is associated with correcting for temperature, quantifying total water surface area, and predicting methane emission factors (see Figure 7 for sensitivity analysis). Time is reported in financial years (1st July to 30th June), as required for the Australian National Inventory Report.

Table S1: Yearly summary of counts, water surface area, methane emissions, and methane fluxes of agricultural ponds in Australia extracted from our model [+/- 95% confidence intervals]. These predictions are reported in the 2024 national greenhouse gas inventory of Australia. Time is reported in financial years (1st July to 30th June), as required for the Australian National Inventory Report.

Financial year	Count (thousands)	Water surface area (kha)	Methane emissions (kt $year^{-1}$)	Methane flux (kg ha^{-1} $year^{-1}$)
1990	1228 [1164; 1317]	114.63 [59.71; 181.84]	26 [5; 119]	227 [28; 1987]
1991	1259 [1194; 1351]	104.88 [54.59; 166.78]	25 [5; 114]	235 [29; 2096]
1992	1296 [1229; 1390]	102.23 [53.23; 161.87]	23 [5; 107]	229 [28; 2012]
1993	1327 [1259; 1424]	127.08 [66.28; 200.19]	29 [6; 134]	231 [29; 2019]
1994	1349 [1280; 1448]	125.54 [65.5; 198.59]	29 [6; 131]	229 [29; 1998]
1995	1388 [1317; 1490]	101.94 [53; 162.76]	23 [5; 108]	228 [28; 2032]
1996	1429 [1356; 1534]	118.42 [61.43; 189.57]	27 [5; 126]	229 [28; 2054]
1997	1458 [1384; 1565]	135.57 [70.59; 216.1]	32 [6; 148]	234 [29; 2090]
1998	1493 [1417; 1604]	113.11 [58.67; 182.33]	27 [5; 132]	241 [28; 2250]
1999	1526 [1448; 1640]	137.01 [71.36; 218.67]	32 [6; 150]	235 [29; 2096]
2000	1555 [1475; 1670]	153.18 [79.8; 243.82]	35 [7; 163]	231 [28; 2037]
2001	1576 [1494; 1694]	144.98 [75.58; 230.05]	34 [7; 160]	237 [29; 2123]
2002	1591 [1509; 1710]	135.22 [70.32; 215.21]	31 [6; 145]	231 [28; 2069]
2003	1617 [1533; 1739]	111.84 [57.94; 178.47]	26 [5; 121]	233 [28; 2090]
2004	1640 [1555; 1764]	136.57 [70.9; 217.79]	33 [6; 153]	238 [29; 2162]
2005	1655 [1569; 1780]	136.75 [71.02; 218.13]	33 [6; 153]	238 [29; 2149]
2006	1663 [1576; 1789]	142.43 [73.98; 226.84]	34 [7; 163]	241 [29; 2204]
2007	1677 [1589; 1804]	112.3 [58.1; 181.35]	27 [5; 127]	237 [28; 2189]
2008	1691 [1602; 1820]	127.84 [66.39; 204.12]	30 [6; 141]	237 [29; 2122]
2009	1696 [1607; 1826]	142.99 [74.36; 229.42]	34 [7; 163]	239 [29; 2189]
2010	1697 [1608; 1827]	142.24 [74; 227.14]	35 [7; 165]	244 [29; 2229]
2011	1704 [1614; 1835]	172.04 [89.6; 274.48]	40 [8; 186]	233 [28; 2071]
2012	1710 [1620; 1842]	175.95 [91.57; 280.76]	41 [8; 189]	233 [28; 2065]
2013	1713 [1623; 1846]	151.22 [78.54; 242.71]	36 [7; 169]	237 [28; 2150]

2014	1720 [1629; 1854]	146.42 [76.05; 234.1]	35 [7; 168]	242 [29; 2211]
2015	1726 [1635; 1861]	152.18 [79.07; 243.61]	37 [7; 175]	241 [29; 2214]
2016	1734 [1642; 1870]	154.49 [80.29; 247.89]	38 [7; 186]	249 [29; 2316]
2017	1740 [1648; 1877]	167.6 [87.14; 267.04]	41 [8; 192]	242 [29; 2209]
2018	1746 [1653; 1884]	127.48 [65.96; 204.12]	31 [6; 150]	245 [29; 2269]
2019	1753 [1659; 1892]	118.99 [61.54; 191]	29 [6; 143]	247 [29; 2316]
2020	1759 [1665; 1899]	114.84 [59.31; 185.03]	27 [5; 127]	234 [28; 2148]
2021	1765 [1671; 1906]	151.85 [78.81; 242.88]	36 [7; 168]	236 [28; 2130]
2022	1771 [1676; 1913]	172.81 [89.86; 276.47]	41 [8; 194]	238 [29; 2157]

Method S1: Methods to run the models presented in the study to inform the 2023 and 2024 national greenhouse gas inventory for Australia.

All necessary data, models, and statistics for replicating our methodology are accessible via the free cloud-based server DEA Sandbox. To access the data and models required to replicate our results, log in to the DEA Sandbox account at https://app.sandbox.dea.ga.gov.au/ (sign up for an account is free).

After logging in with the default configuration (16 Gb), open a Python Notebook and clone the GitHub repository including all data and codes, typing:
!git clone https://github.com/DPIRD-DMA/Weather-to-water.git

This command will create a new folder in your home directory called “Weather-to-water” (see screenshot below).

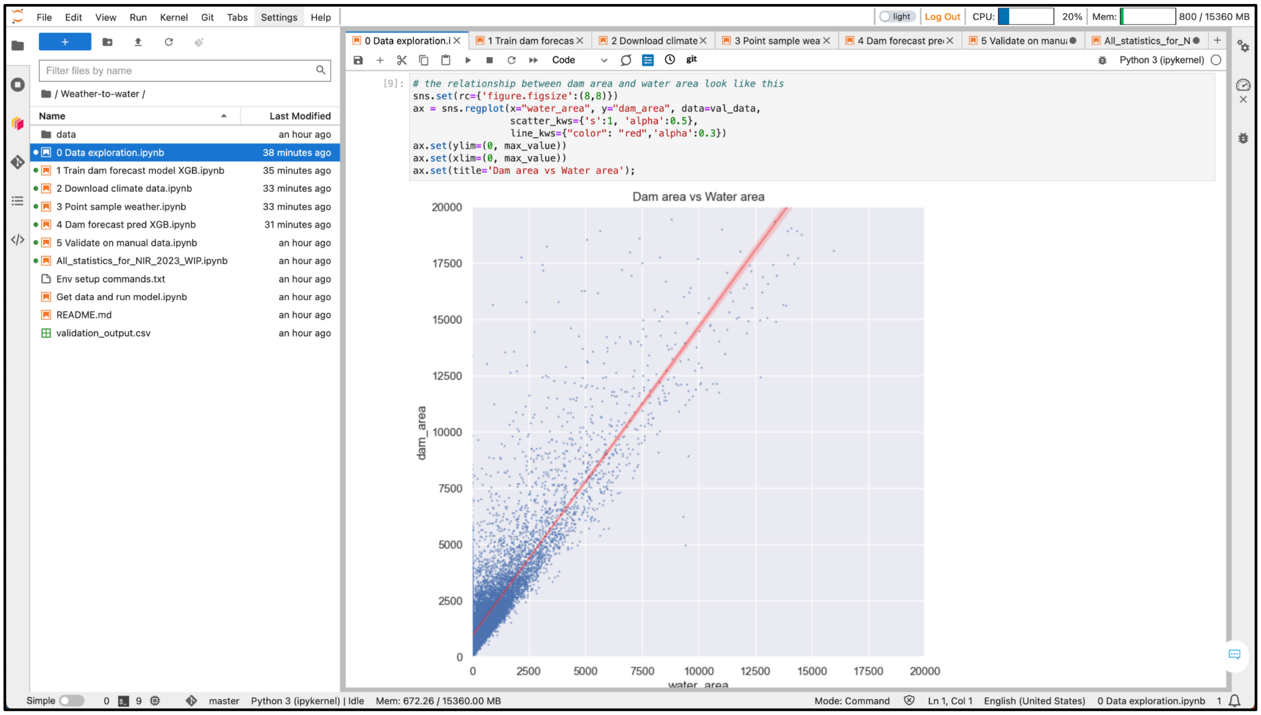

The model is executed in Python and organised in six Jupyter notebooks. Each notebook is commented and presents different parts of our approach.

The first notebook (0 Data exploration.ipynb) presents the model for identifying agricultural ponds and quantifying their water surface.

The second notebook (1 Train dam forecast model XGB.ipynb) trains the Extreme Gradient Boosting (XGBoost) regression model using monthly weather data to predict pond water capacity.

The third notebook (2 Download climate data.ipynb) downloads the climate maps for monthly total rainfall and average temperature for Australia since 1990.

The fourth notebook (3 Point sample weather.ipynb) extracts the monthly time series for rainfall and temperature at the location of each agricultural pond using climate maps.

The fifth notebook (4 Dam forecast pred XGB.ipynb) uses the calibrated XGBoost with monthly time series of local rainfall and temperature to predict water surface dynamics for each agricultural pond.

The sixth notebook (5 Validate on manual data.ipynb) validates the XGBoost with manually collected data.

The seventh notebook (6 Statistics for NIR.ipynb) compiles monthly methane emissions from agricultural ponds, adjusts for current and historical densities of Australian ponds, and calculates total yearly methane emissions.
